# Prospects and challenges of imaging neuroinflammation beyond TSPO in Alzheimer’s disease

**DOI:** 10.1007/s00259-019-04462-w

**Published:** 2019-08-08

**Authors:** Delphine Boche, Alexander Gerhard, Elena Rodriguez-Vieitez, on behalf of the MINC Faculty

**Affiliations:** 1 Clinical Neurosciences, Clinical and Experimental Sciences Academic Unit, Faculty of Medicine, University of Southampton, Southampton, UK; 2 Department of Nuclear Medicine and Geriatric Medicine, University Hospital Essen, Essen, Germany; 3 Wolfson Molecular Imaging Centre, University of Manchester, Manchester, UK; 4 Department of Neurobiology, Care Sciences and Society, Division of Clinical Geriatrics, Karolinska Institutet, Stockholm, Sweden

**Keywords:** TSPO, Microglia, Astrocyte, Alzheimer’s disease, PET, MRI

## Abstract

Neuroinflammation, as defined by the activation of microglia and astrocytes, has emerged in the last years as a key element of the pathogenesis of neurodegenerative diseases based on genetic findings and preclinical and human studies. This has raised the need for new methodologies to assess and follow glial activation in patients, prompting the development of PET ligands for molecular imaging of glial cells and novel structural MRI and DTI tools leading to a multimodal approach. The present review describes the recent advancements in microglia and astrocyte biology in the context of health, ageing, and Alzheimer’s disease, the most common dementia worldwide. The review further delves in molecular imaging discussing the challenges associated with past and present targets, including conflicting findings, and finally, presenting novel methodologies currently explored to improve our in vivo knowledge of the neuroinflammatory patterns in Alzheimer’s disease. With glial cell activation as a potential therapeutic target in neurodegenerative diseases, the translational research between cell biologists, chemists, physicists, radiologists, and neurologists should be strengthened.

**Electronic supplementary material:**

The online version of this article (10.1007/s00259-019-04462-w) contains supplementary material, which is available to authorized users.

## Introduction

There is increasing evidence that inflammation plays a key role in the development and progression of neurodegenerative diseases such as Alzheimer’s disease (AD), likely being involved in its etiology and as a disease-promoting factor [1, 2]. Indeed, accumulating experimental, genetic, and epidemiological data implicate the innate immune system in AD, most prominently involving genes for immune receptors or immune-related functions such as *APOE* (apolipoprotein E), *CR1* (complement receptor 1), *B1N1* (bridging integrator 1), *CD33*, *TREM2* (triggering receptor expressed on myeloid cells 2), and *PICALM* (phosphatidylinositol binding clathrin assembly protein). These new developments have raised the interest in investigating glial cells towards understanding the origin of AD and to design novel disease-modifying therapies.

Neuropathologically, AD is defined by the aggregation and deposition of amyloid-β (Aβ) plaques in the parenchyma and hyperphosphorylated tau in neurons, and therefore, AD has been mostly conceptualised as a proteinopathy. The majority of clinical trials have aimed at clearance of these protein forms, however, with very limited success clinically [3]. While longitudinal neuroimaging studies using PET (positron emission tomography) tracers for Aβ and tau suggest that Aβ precedes tau pathology [4], the mechanistic link between these two pathological features is not well known. Interestingly, new preclinical data suggest that neuroinflammation might be a possible link between Aβ and tau proteinopathies. In particular, Aβ aggregation may trigger activation of microglia and astrocytes, the cells of the brain immune system, leading to the release of neuroinflammatory markers that might be contributing to tau pathology and spreading [5, 6]. These studies have shown that microglia activation can lead to astrocyte activation and vice-versa, but the regional and temporal patterns of astrocytosis and microgliosis in relation to other pathophysiological changes are not well known. These recent findings stimulate further research on neuroimaging of microglia and astrocytes using PET tracers in combination with other imaging modalities and fluid biomarkers.

## Microglia in the healthy brain

Microglia migrate from the yolk sac [7] and colonise the brain early during embryogenesis to form the resident immune cells of the brain. Their maintenance and expansion in the central nervous system (CNS) relies exclusively on their capacity of self-renewal without any further colonisation [8–10]. The population remains stable over the lifetime with a slow turnover [10], of an approximate lifespan of 4 years in humans [11], and they are the main identified proliferating cells in the human brain from the age of 3 years old [12]. Microglia are defined by a unique molecular signature, known as their *homeostatic signature*, which is driven by the expression of the transforming growth factor (TGF)-β1 cytokine, differentiating them from the other brain cells and myeloid populations [13]. This signature encompasses expression of a specific set of genes reported in Table 1.

**Table 1:** Microglial markers identified from rodent models and human studies

Phenotype	Mouse (*gene*, protein)	Human (*gene*, protein)	Morphology [142]
Homeostatic, physiological status [13–16, 143]	*Tgfbr1, Smad3, C1qa, C1qb, Cst3, Csf1r, Ctsd, Ctss, Cx3cr1, Entpd1 Fcrls, Hexb, Olfml3, P2y12, Tmem119, Tmsb4x, Sparc*	TMEM119 P2Y12 CX3CR1 *Fclrs, C1qa, Pros1, Mertk, Gas6* Iba1	Ramified with fine processes
Ageing [17, 18]	MHC class II Complement CR3 (CD11b) CD68, CD11c TLR members CD86	FcγRs (CD64, CD32, CD16b) MHC class II (*HLA* subtypes, HLA-DR) Complement (*C1qA, C1qB, C1qC, C1s, C3, C3AR1, C4, C5, C5AR1*), *C1QBP*, *CFH, CFHR1, clusterin* TLR members (, *TLR2*, *TLR4*, *TLR5*, *MYD88*) *Calprotectin (S10A8, S100A9)*, CD14 *IRAK3, SOCS3* Inflammasome (*Casp1*, *TXNIP, PANX1, PANX2*) Chemokines (*CCR1, CXCL5, CXCL16*) CD68, CD11b	Ramified Less ramified with shorter and thicker processes
Alzheimer’s disease [14–17, 19]	MGnD profile *Apoe,Trem2,Tyrobp,Axl,Clec7a, Ccl2,Csf1,Itgax,Lilrb4, Lgal3, Gpnmb, Fabp5, Spp1* DAM profile *Apoe,Trem2,Tyrobp,Axl, B2m, Fth1, Lyz2, Ctsb, Ctsd, Ctsl, Cst7,Csf1, Ccl6, Lpl, Cd9,Itgax,Clec7a,Lilrb4, Timp2*	FcγRs (CD64, CD32b, CD16b) MHC II (HLA-DR, DP, DQ) Complement (*C4*, *C3AR1, C5AR*1) *S100A8* *CSFR1* Chemokines (*CXCL16*, CCR2) TLR (*TLR2, TLR4, TLR5, TLR7*) CD68, CHI3L1, IL4R, MSR-A	Ramified Less ramified with shorter and thicker processes Ameboid shape (round shape with no processes)

Underlined, common genes identified in both mouse microglial profiles

*Apoe*, Apolipoprotein; *Axl*, Tyrosine-protein kinase receptor; *B2m*, beta-2 microglobulin; *C1qA*, Complement C1q subcomponent A; *C1QBP*, C1q binding protein; *C3AR1*, Complement component 3a receptor 1; *C5AR1*, Complement component 5a receptor 1; *Casp1*, caspase 1; *Clec7*, C-type lectin domain family 7; *CCR*, Chemokine (C-C motif) receptor type; *CHI3L1*, Chintinase-3 Like-1; *CXCL*, Chemokine (C-X-C motif) ligand; *Cx3cr1*, CX3C chemokine receptor 1 (fractalkine receptor); *CFH*, Complement Factor H; *CFHR1*, Complement Factor H Related 1; *CR3*, Complement receptor 3; *Cst3*, Cystatin; *Ctsd*, Cathepsin D; *Ctsl*, Cathepsin L; *Ctss*, Cathespin S; *Csf1r*, Colony-stimulating factor 1 receptor; *Entpd1*, Ectonucleoside triphosphate diphosphohydrolase 1; *Fabp5*, Fatty acid binding protein 5; *FcγR*, Fcγ receptor; *Fclrs*, Fc receptor-like S; *Fth1*, Ferritin heavy chain 1; *GAS6*, growth arrest-specific 6; *Gpnmb*, Glycoprotein Nmb; *GPR34*, G protein-coupled receptor 34; *HLA*, Human leucocyte antigen; *Hexb*, Beta-hexosaminidase subunit beta; *Iba1*, Ionised calcium-binding adaptor molecule 1; *IRAK3*, Interleukin 1 receptor associated kinase 3; *Itgax*, Integrin alpha X; *IRAK3*, Interleukin-1 receptor associated kinase 3; *Lilrb4*, Leukocyte Immunoglobulin Like Receptor B4; *Lgal3*, Galectin-3; *Lpl*, lipoprotein lipase; *Lyz2*, Lysozyme 2; *MERTK*, C-mer proto-oncogene tyrosine kinase; *MHC*, Major histocompatibility complex; *MSR*, Macrophage scavenger receptor; *MYD88*, myeloid differentiation primary response 88; *PAX*, Pannexin; *Olfml3*, Olfactomedin Like 3; *P2y12*, Purinergic receptor P2Y12; *Pros1*, Protein S; *S100A8*, S100 calcium-binding protein A8; *Smad3*, SMAD family member 3; *SOCS3*, Suppressor of cytokine signalling 3; *Sparc*, Secreted protein acidic and cysteine rich; *Spp1*, Secreted phosphoprotein 1; *Timp2*, Metallopeptidase inhibitor 2; *Tgfbr1*, Transforming Growth Factor Beta Receptor 1; *TLR*, Toll-like receptor; *TMEM*, Transmembrane protein; *Tmsb4x*, Thymosin Beta 4 X-Linked; *Tyrobp*, TYRO Protein Tyrosine Kinase Binding Protein; *Trem2*, triggering receptor expressed on myeloid cells 2; *TXNIP*, Thioredoxin-interacting protein

**Fig. 1:**
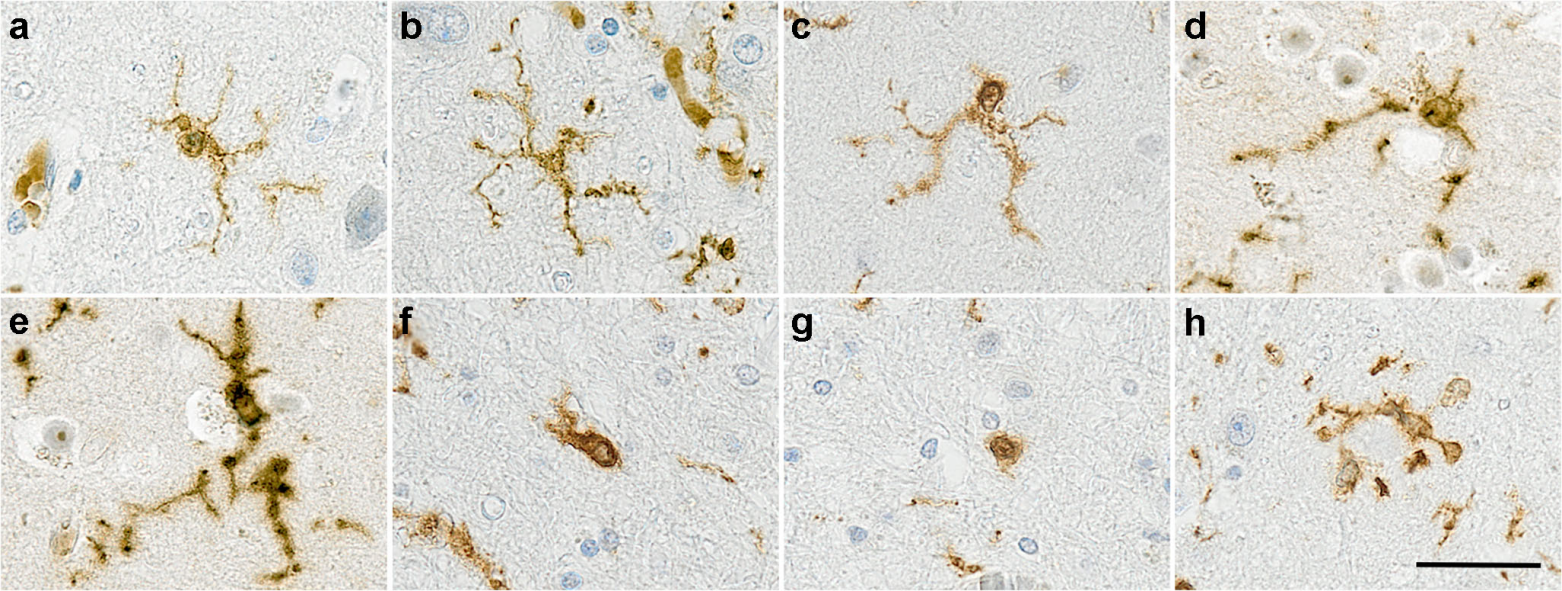
Illustration of different microglial morphologies in human brain identified with Iba1. (**a-g**) From ramified to ameboid microglia; (**h**) cluster of microglia around amyloid plaques as observed only in Alzheimer’s disease. Counterstaining Haematoxylin, scale bar = 30 μm

In humans, microglia represent 0.5 to 16% of the total brain cells [20], with significantly more cells in the white than in grey matter. They have a ramified morphology having multiple fine processes (Fig. 1a-d), and time-lapse imaging experiments in mice have demonstrated that microglia are extremely motile cells, screening constantly the parenchyma via their processes [21]. During brain development, microglia participate in the wiring and maturation of the brain [22] by pruning excessive synapses [23] and influencing neurogenesis [24]. In adult, microglia survey and sense any changes in order to respond rapidly to any insult or injury [21], and participate in synaptic communication [25, 26]. The properties of sensing and interacting with their micro-environment are performed via microglia-specific transcripts termed the *microglial sensome* [27] with several of the sensome genes identified later as components of the homeostatic signature such as *P2Y*_*12*_ [13, 27]. It is assumed that similar functions are performed by microglia in humans.

Ageing per se predisposes to peripheral inflammation, a concept known as “inflammaging” [28], and this concept is supported by the presence of altered mRNA expression and proteins of inflammation-related genes in the middle-aged human and mouse brain [29] (Table 1). In aged mice, the expression of the sensome genes is diminished [27]. Interestingly, the major changes of the expression profiles of immune- and inflammation-related genes occur during the course of cognitively normal ageing rather than disease conditions and involve upregulation of genes coding for inflammasome signalling, Fcγ receptors, and HLA [17]. Morphological microglial changes characterised by reduced branching and arborised area have been described in humans [30] (Fig. 1e-g) suggesting that microglia become dysfunctional or senescent with ageing. These molecular and morphological changes imply that with age, microglia lose their neuroprotective properties associated with chronic neurodegeneration [31]. A recent study investigating the phenotype of aged human microglia highlighted that microglial ageing manifests as loss of function as well as gain of function changes to give a unique aged-related microglia phenotype [32]. This profile was enriched in susceptibility genes for AD but interestingly, independent from APOE4, the main risk factor for AD.

A number of concepts related to the phenotypes that microglia can acquire have emerged from experimental models. Microglial priming is defined as a prolonged and exaggerated immune challenge resulting from an acute inflammatory event in an ongoing inflammatory environment [33]; whereas innate immune memory is associated to cell reprogramming following a primary immune stimulus that leads to increased (trained) or decreased (tolerant) responses to a secondary inflammatory stimulus [34]. Primed or reprogrammed microglia have an enhanced response to a second stimulus. Therefore, an integrated nomenclature under the term of microglial memory was recently proposed to encompass both phenotypes [34] (Table 2). These concepts are important and highly relevant to the lifetime of infections in humans and to the microglial lifespan with some of cells reported to be more than 20 years old [11], and thus capable of remembering the inflammatory events [19, 35]. Indeed, even in the absence of brain injury, insult, or infection, it is now acknowledged that systemic immunity will affect the brain immunity [18]. However, the relevance of the rodent experiments to humans is still unclear. To summarise, microglia are adaptive cells with the phenotype and morphology determined by their local environment (Table 1).

**Table 2:** Definitions of the different microglial phenotypes

Status	Definition
Physiological/homeostatic	Microglia in an immune stimulus-free environment.
Primed microglia (one immune stimulus)	Prolonged and exaggerated increased immune response due to microglia already activated by an initial event.
Trained microglia (repetitive immune stimuli)	Increased microglial responses following priming.
Immune memory	Long-term consequences of trained microglia inducing cell reprogramming. This leads to either increased (primed) or decreased (tolerant) immune responses.

## Microglia in Alzheimer’s disease

Genome-wide association studies have highlighted variation in genes of the innate immunity as risk factors for AD, emphasising the role of microglia, not only in responding to the neurodegeneration, but also in the onset and progression of the disease. Two microglial phenotypes have been determined from mouse AD models. The *Disease-Associated Microglia* (DAM) is characterised by low expression of homeostatic markers (e.g. *CX3CR1*, *P2y12*, *Tgfb1*) and elevated levels of lipid metabolism, phagocytosis, apoptosis, and AD-associated genes (e.g. *APOE*, *Trem2*) [15]. Similarly, the *Microglial Neurodegenerative Phenotype* (MGnD) is driven by a rise in APOE and apoptotic markers (e.g. *Axl*, *Clec7a*), and the fading of the microglial homeostatic signature [16] (Table 1). Both profiles are consistent with microglia responding to and participating in the ongoing neurodegeneration, and thus are found in any mouse models of neurodegenerative diseases characterised by neuronal loss [16]. Therefore, it is unclear to what extent microglia promote or respond to neurodegeneration, likely both, and we still do not have insight into how microglia participate in the onset of the disease.

**Fig. 2:**
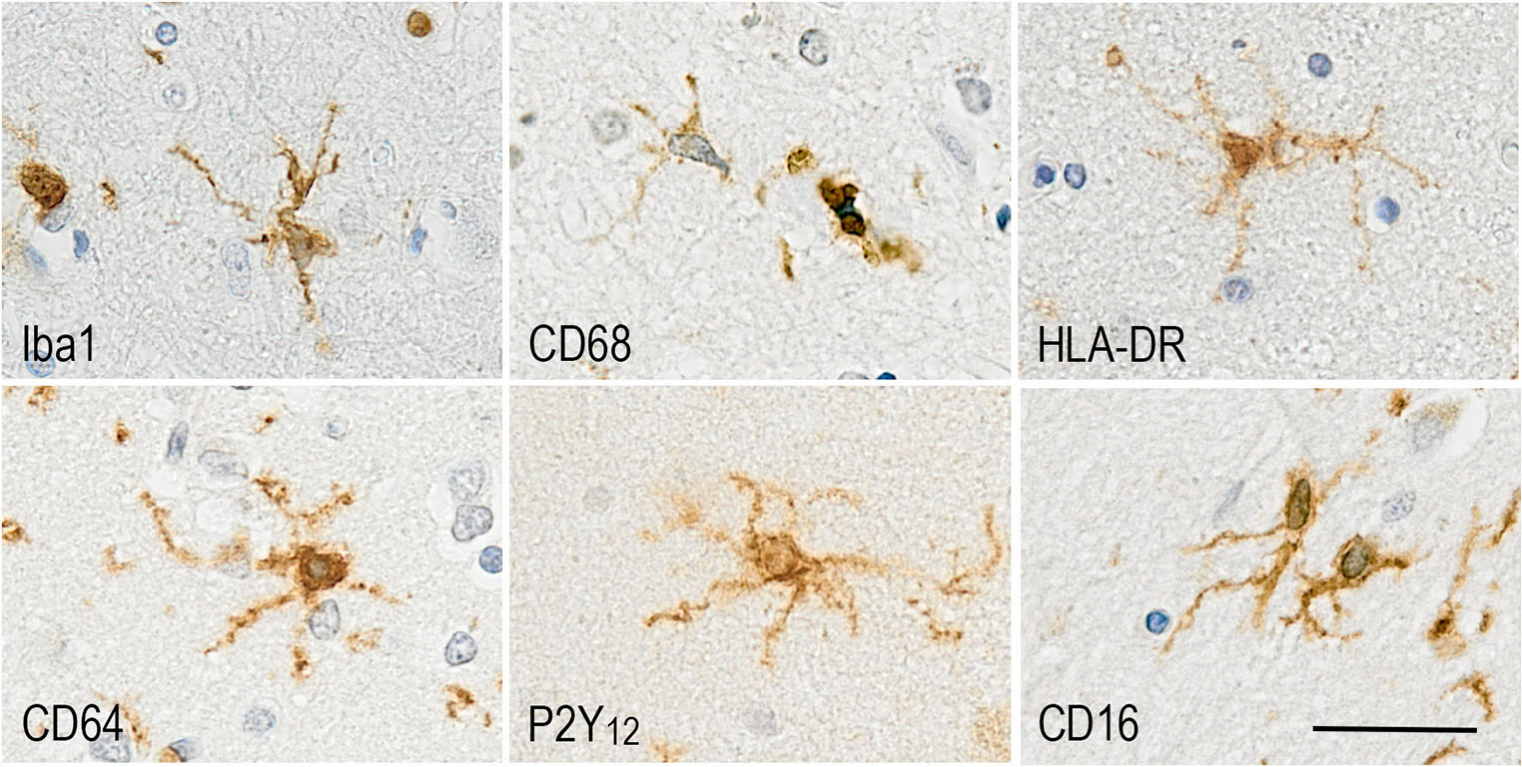
Microglial proteins expressed in human brain and associated with different functions: Iba1 (Motility); CD68 (phagocytosis); HLA-DR (antigen presentation); CD64 (FcγRI high-affinity activating receptor reflecting presence of immunoglobulins); P2Y_12_ (homeostasis); CD16 (FcγRIII low-affinity activating receptor for immune complexes). Counterstaining Haematoxylin, scale bar = 30 μm

In humans, microglial activation is a neuropathological feature of the disease and microglia participate to the formation of the neuritic plaques, clustering around Aβ deposits (Fig. 1h). Using a mathematical model, activated microglia have been placed after Aβ plaques but before tau pathology [6]. However, longitudinal studies of PET imaging using microglia, Aβ, and tau ligands are necessary to confirm in vivo the dynamics of these events. In an unbiased elderly population, immunophenotyping of microglia using markers associated with specific microglial functions (Fig. 2) has revealed that in AD, microglia appear to lose motility-associated proteins (e.g. Iba1), a key function in synaptic support [36] and change towards a more phagocytic phenotype (CD68, macrophage scavenging receptor (MSR)-A), partly driven by the APOE genotype [14]. This study highlighted that microglia were responding differently to Aβ and tau in participants with or without dementia and were able to adopt different functions relatively independently, emphasising the likely coexistence of different microglial populations within the same brain. Interestingly, the brain environment reveals inflammatory heterogeneity, with a mixture of pro- and anti-inflammatory compounds, observed *post-mortem* in late stages of the disease [19, 37]. However, in the early stages of AD, a more polarised inflammatory environment towards either pro- or anti-inflammatory profile was reported to be associated with different pathological severity [37], possibly reflecting different stages of the disease or different microglial populations.

The immune reactions are clearly complex in AD, with evidence of temporal changes of microglia. However, these studies lack information on the specific functions performed by microglia throughout the course of the disease. Microglia activation undergoes various stages, which have been traditionally classified into pro-inflammatory/detrimental (M1) or anti-inflammatory/protective (M2). However, there is increasing evidence that the M1/M2 division is somewhat artificial and even considered obsolete. Instead, microglia assume a continuum of activation states characterised by the expression of multitude of markers that overlap between M1 and M2 states [38], continuum which can evolve, potentially driving, the disease [37]. This complexity of microglial activation represents one of the main challenges to identify targets suitable for molecular imaging.

## Molecular imaging targets for microglia in Alzheimer’s disease and mild cognitive impairment (MCI) patients

**Fig. 3:**
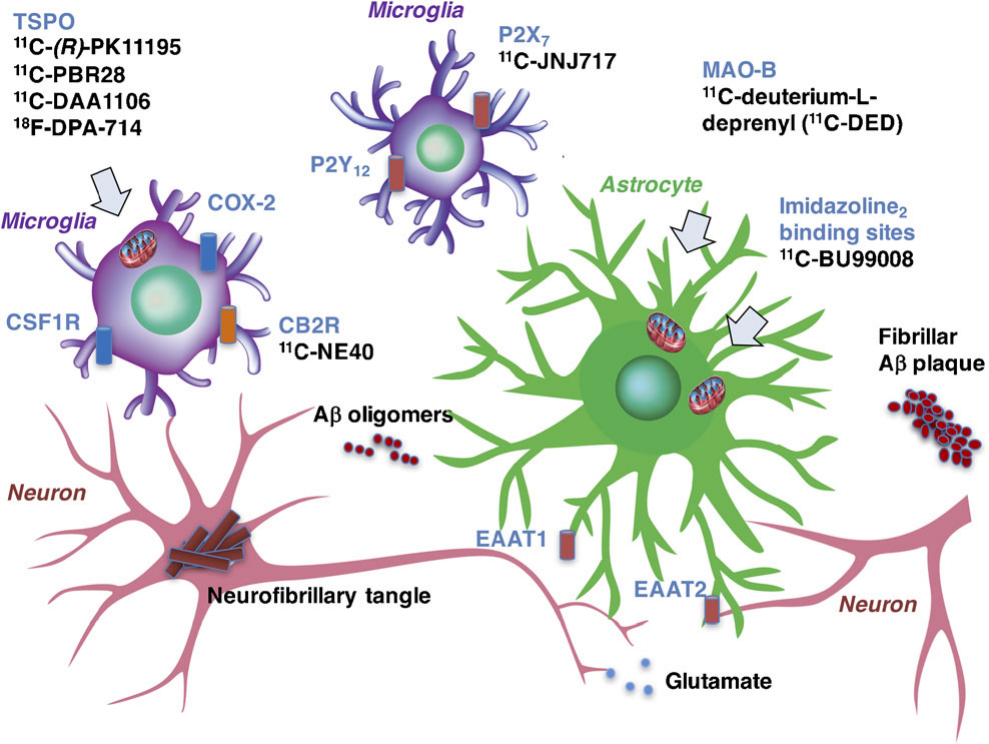
Cartoon illustrating emerging targets and PET tracers for the selective in vivo visualisation of activated microglia and astrocytes in Alzheimer’s disease

Molecular imaging studies in AD have largely focused on visualising activated microglia, most commonly measured by elevated expression of translocator protein 18 kDa (TSPO), a five transmembrane domain protein mainly located in the outer mitochondrial membrane of microglia [39]. While mostly considered as a specific marker of microglial activation, TSPO has been found to be overexpressed in activated astrocytes [40, 41]. One of the limitations associated with the use of TSPO is its inability to distinguish between the different phenotypes expressed by microglia, potentially lacking disease-specificity. These limitations have led to the search for alternative biological targets for imaging microglia. Some of the emerging tracers target P2X_7_ receptors, cannabinoid receptor type 2 (CB2), cyclooxygenase (COX)-2, colony-stimulating factor 1 receptor (CSF1R), and reactive oxygen species (reviewed in [39, 42], Fig. 3). Although progress has been made, most of the emerging tracers target only the pro-inflammatory (M1) phenotype of activated microglia, and several tracers have issues of limited specificity as they bind also to other cells such as astrocytes and endothelial cells. Among the most promising novel tracers are those that target purinergic receptors including P2X_7_, selective towards the M1 phenotype and overexpressed in AD brain, and P2Y_12_, selective towards the M2 phenotype and with reduced expression around Aβ plaques in AD brain [42]. Of note, P2Y_12_ PET tracers are yet the only ones identified to target the M2 anti-inflammatory phenotype, which has been associated with physiological/homeostatic microglia.

Below, we will review the human imaging studies aiming to characterise in vivo microglial activation in MCI and AD dementia patients, both with TSPO and non-TSPO tracers.

### PET studies using TSPO tracers

When reviewing the work done on using PET tracers for TSPO, the literature shows divergent and sometimes conflicting results. This is partially due to different binding properties of the various PET tracers but also due to methodological issues when quantifying the PET signal.

The ^11^C-labelled (*R*)-isomer of PK11195 is the best-characterised PET ligand for imaging activated microglia in vivo. In a pilot PET study in eight AD patients (median age 63.5 years, median MMSE 19.5) vs. 15 control subjects, increased ^11^C-(*R*)-PK11195 binding potential (BP) was measured in the inferior and middle temporal gyri, fusiform gyri, left parahippocampal gyrus, amygdala, and posterior cingulate [43]. A subsequent study examined ^11^C-(*R*)-PK11195 and ^11^C-Pittsburgh compound B (^11^C-PiB, a ligand for Aβ) BP in 13 AD patients (mean age 65.6 years, mean MMSE 21.2) using both region-of-interest (ROI) and voxel-based analysis. Significant increases in ^11^C-(*R*)-PK11195 BP were found in frontal, temporal, parietal, and occipital association cortices, cingulate cortex, and striatum, in a pattern that corresponded with areas of increased ^11^C-PiB binding [44], connecting microglial activation to Aβ plaque deposition. Interestingly, ^11^C-(*R*)-PK11195 binding in the posterior cingulate, frontal, and parietal cortices, but not ^11^C-PiB uptake ratio, was negatively correlated with MMSE [44]. This implied that microglia rather than Aβ deposition was the culprit for the cognitive decline, before the confirmation by the genetics of a role of the innate immunity in AD pathogenesis [45]. These findings were replicated by others [46]. Another study using the alternative TSPO tracer ^11^C-DAA1106 [47] also detected increased signal in AD patients in all regions investigated but did not assess the link with the cognitive decline.

**Fig. 4:**
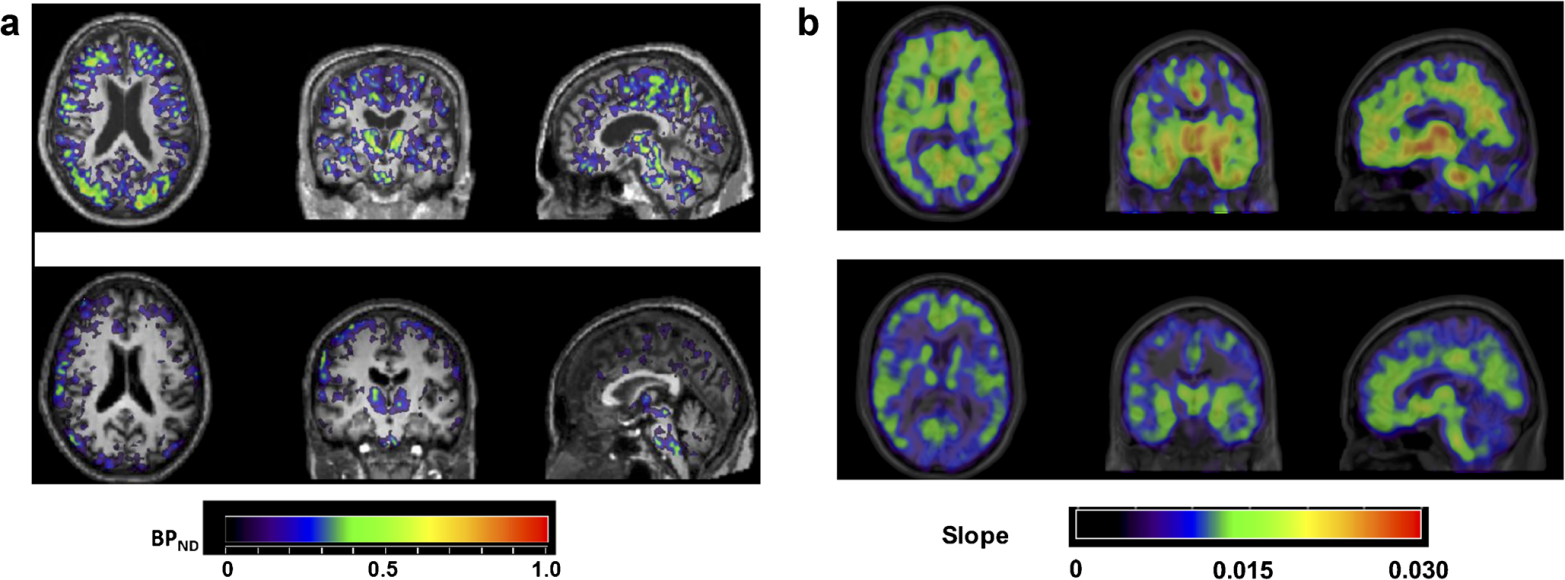
**a** Parametric ^11^C-*(R)*-PK11195 PET images of microglial activation in one MCI patient (upper row) and one healthy volunteer (lower row); PET images are displayed on each subject’s respective T1-weighted MRI scan normalised to the SPM5 T1 brain template and the colour bar indicates non-displaceable binding potential (BPND) values. **b** Parametric ^11^C-deuterium-L-deprenyl PET images of astrocyte activation in one Aβ-positive MCI patient (upper row) and one healthy volunteer (lower row); PET images are displayed on each subject’s respective T1-weighted MRI scan normalised to the SPM5 T1 brain template and the colour bar indicates modified-reference (cerebellar grey matter) Patlak slope values; the ^11^C-deuterium-L-deprenyl PET imaging data were kindly provided by Prof. Agneta Nordberg, Karolinska Institutet, Sweden

Assessment of microglial activation has also been of particular interest in patients with MCI to aim at exploring the kinetic and temporal association of the different neuropathological features. In our own group study of 12 MCI compared with 7 healthy volunteers, ROI analysis revealed increased ^11^C-*(R)*-PK11195 binding in the posterior cingulate gyrus, parietal, occipital, frontal, and temporal lobes as well as in the putamen (Fig. 4a) (A. Gerhard, unpublished results). Interestingly, in this series, the MCI patients that were either homo- or heterozygote for the ApoE ε4 allele seemed to show higher binding than the ones with an ApoE ε3/3. Our study links two risk factors for AD, immunity, and APOE genotype. Study of ^11^C-(*R*)-PK11195 and ^11^C-PiB PET in 14 patients with amnestic MCI (mean MMSE 27.7), 22 with AD (mean MMSE 21.5), and 10 controls (mean MMSE 29.9) identified increased ^11^C-(*R*)-PK11195 BP in frontal cortex of PiB-positive MCI patients [48], but also observed MCI patients with either increased PK11195 or PiB signal. The authors propose microglial activation and Aβ deposition as two independent processes in AD. This might be the case; however, we can argue that the discrepancy, especially in the PiB-negative MCI patients, might be the consequence of different processes with some leading to another dementia than AD. Nevertheless, a recent study confirmed the spatial association between the ^11^C-*(R)*-PK11195 binding and cortical Aβ load as measured with ^11^C-PiB in 42 MCI patients, with an overlapped distribution [49].

In contrast to the above-listed positive studies, a study of six MCI patients (mean MMSE 28), six AD patients (mean MMSE 19), and five controls failed to find any differences in ^11^C-(*R*)-PK11195 binding using ROI analysis, even when ^11^C-PiB uptake was taken into account [50]. A further study failed to identify any significant group differences in PK11195 binding between controls, AD, and MCI patients using both ROI-based and voxel-based analysis approaches [51]. Therefore, it is not yet clear whether the disparities in the findings between the studies are due to differences in methodology, or in the selection and clinical stage of patients.

As mentioned above, while PK11195 is thought to bind predominantly to activated microglia, increases in binding to activated astrocytes have been reported by some authors [52]. However, ^3^H-(*R*)-PK11195 binding in *post-mortem* tissue from patients with neuroinflammatory disorders correlated strongly with CD68-phagocytic microglia, but not GFAP-positive astrocytes, albeit with a lower binding affinity compared to the novel TSPO ligand ^3^H-DAA1106 [53]. A further potential limitation is that in vivo studies with ^11^C-(*R*)-PK11195 generally display a relatively low signal-to-noise ratio [54].

The most appropriate method of analysis for PET data in neurodegenerative disorders is also the subject of debate, as the selection of a reference region is difficult due to constitutive expression of TSPO within the brain and potentially very widespread pathology. Modified methods using kinetic analysis to identify a reference tissue cluster have been employed in the analysis of ^11^C-(*R*)-PK11195 PET data in neurodegenerative disease [55]. Lastly, as a ^11^C-labelled tracer, ^11^C-(*R*)-PK11195 has only a short half-life and necessitates a cyclotron on site for tracer production, limiting its use. These limitations have led to the development of novel TSPO ligands for PET imaging.

Over the last years, numerous novel radiotracers for activated microglia have been developed [56]. The compounds DAA1106 and PBR28 have been shown to have 10-fold higher in vitro affinity for TSPO compared to PK11195 [57, 58]. ^11^C-DAA1106 has been shown to have higher BP in prefrontal, parietal, anterior cingulate, and occipital cortex, striatum, and cerebellum in a PET study of 10 AD patients vs. 10 healthy controls, although partial volume effects could explain some of these changes [47]. A further study in seven MCI patients, 10 AD patients, and 10 healthy controls showed increased ^11^C-DAA1106 BP in temporoparietal cortex, anterior cingulate, and striatum in both MCI and AD patients compared to controls [59]. However, in contrast to the previous studies using PK11195, no difference in the DAA1106 binding was observed between the MCI and AD patients. This might be the consequence of the finding of populations of high- and low-affinity binders for TSPO ligands other than ^11^C-(*R*)-PK11195 complicating further the analysis of the data [58]. Thus, studies utilising such ligands require analysis of TSPO binding affinity to correct for this factor. Of note, early-onset (< 65 years) patients were detected to have greater binding of the ^11^C-PBR28 ligand than late-onset patients in parietal cortex and striatum, and ^11^C-PBR28 binding correlated with lower age of onset [60], adding to the complexity of the interpretation of the TSPO data and our understanding of the role of microglia in AD.

Attempts at exploring the time course of microglial activation was performed in a large prospective study including 64 patients with AD (prodromal AD *n* = 38; AD dementia *n* = 26) and 32 controls. A simple ratio method was used with cerebellar grey matter as reference tissue to measure TSPO binding. The study showed that microglial activation appears at the prodromal and possibly at the preclinical stage of AD, and the authors concluded that microglia might play an early protective role in the clinical progression of the disease [61]. Subsequently the authors followed a proportion of the participants over 2 years and found that high initial ^18^F-DPA-714 binding was correlated with a low subsequent increase of microglial activation and favourable clinical evolution, whereas the opposite profile was observed when initial ^18^F-DPA-714 binding was low, independently of disease severity at baseline. They felt that their results support a pathophysiological model involving two distinct profiles of microglial activation signatures with different dynamics, which differentially impact on disease progression and may vary depending on patients rather than disease stages [62]. However, without the use of ligands associated with already identified profiles of microglia, as described previously, this remains speculative.

Only further clinical evaluations and direct comparisons with ^11^C-(*R*)-PK11195 will show whether any of the 2nd- and 3rd-generation TSPO tracers are indeed superior in the clinical context. As there are known to be species differences in TSPO binding, in vitro and preclinical animal data do not always directly translate into clinical applications in humans [56].

### PET studies using non-TSPO tracers for neuroinflammatory changes in AD

It is important to realise that TSPO expression is only one phenomenon within the complex process of microglial activation and currently effort goes into the development of in vivo imaging markers that can measure other pathways of this process. Developing and evaluating PET ligands is a lengthy process and currently only very few novel non-TSPO ligands are being used in a clinical context.

The cannabinoid type 2 receptor (CB2R) is expressed by immune cells including monocytes and macrophages. In the brain, CB2R is primarily found on microglia. Its upregulation has been reported in animal models of AD, and ^11^C-NE40—a tracer for CB2R—has been evaluated in healthy controls and patients with AD. Surprisingly, AD patients showed overall significantly lower CB2R binding than healthy controls and no relationship was found between regional or global Aβ load and CB2R availability, possibly due to lower affinity or selectivity of ^11^C-NE40 to CB2R than to CB1R [63].

^11^C-JNJ717, a selective P2X_7_ receptor tracer, one of the ATP-gated ion channels expressed on activated microglia, was evaluated in healthy volunteers and Parkinson’s disease (PD) patients where microglia are known to become activated as part of the neuropathological changes. No difference was found in binding between healthy controls and PD patients [64]. So far, no clinical studies have been conducted using this tracer in AD.

The colony-stimulating factor 1 receptor (CSF1R) might also be a very interesting target for neuroinflammation imaging and very recently preclinical data in a murine model of AD have been acquired [65], but no clinical studies have been conducted as of yet.

## Astrocytes in the healthy brain

**Fig. 5:**
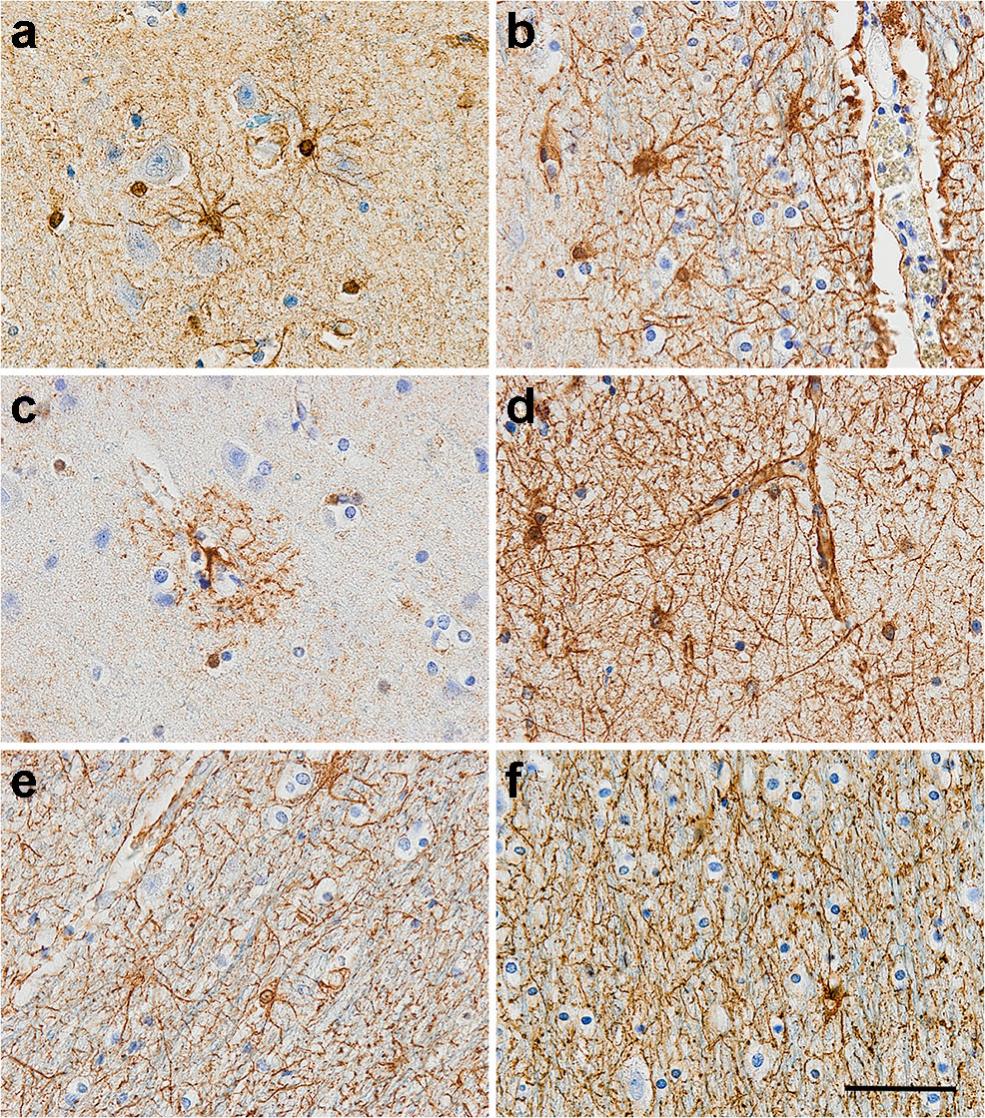
GFAP-positive astrocytes in human brain observed (**a**, **b**) in the grey matter; (**c**, **d**) with the endfeet of the processes forming a component of the blood-brain barrier; and (**e**, **f**) in the white matter. Counterstaining: Haematoxylin, scale bar = 50 μm

Astrocytes derive from heterogeneous populations of progenitor cells in the neuroepithelium. They adopt a star-like morphology and in humans, form 20 to 40% of all glia [66]. Several populations of astrocytes cohabit in the adult brain [67] (Fig. 5). In the grey matter, we find (i) the protoplasmic astrocytes characterised by highly branched bushy processes and located in the cortical grey matter; (ii) the primate-specific interlaminar astrocytes located in the superficial cortical layers I and II of the cortex; and (iii) the varicose projection astrocytes in layers V and VI [67, 68]. The fibrous astrocytes are mainly present in the white matter along the myelinated axons and exhibit long and straight processes [67].

Astrocytes are key elements of brain homeostasis displaying numerous physiological functions. They are involved in (i) neurodevelopment [69]; (ii) synaptic function [70]; (iii) energy metabolism [71]; (iv) the neurovascular unit via their processes [72]; and recently, they were identified to be involved in (v) the circadian clock [73]. This large functional diversity is in keeping with the heterogeneous and pleomorphic astrocytes population throughout the brain. Glial fibrillary acidic protein (GFAP), an intermediate filament and major component of the astrocyte skeleton, is the most common marker to identify astrocytes, with its expression higher in fibrous than protoplasmic astrocytes [67]. However, it is now acknowledged that GFAP does not detect all astrocytes [74]. Other markers, based on the astrocyte functions, have been utilised sometimes in combination, to identify the different populations in humans including the enzyme aldehyde dehydrogenase 1 family member L1 (Aldh1l1), calcium-binding protein S100B, the glutamate transporters excitatory amino-acid transporter (EAAT) 1 and 2 for the synaptic transmission, the enzyme glutamine synthetase, and the Aquaporin 4 expressed at astrocyte endfoot processes to regulate water homeostasis [75]. Novel astrocyte-specific markers are emerging from rodent studies such as CD44 [76], CD51 (integrin alpha V protein), CD63 (LAMP-3), and CD71 (transferrin receptor 1) [77].

Ageing modifies astrocytes including their morphology with the processes becoming shorter and thicker, increasing cell density and GFAP expression [78], together considered as common features of activated astrocytes. Therefore, this suggests that, like microglia, astrocytes become reactive with age. The molecular profile of aged astrocytes, defined from rodent brain, encompasses upregulation of the complement system (*C3* and *C4B*); major histocompatibility complex (*H2-D1* and *H2-K1*); cytokines/chemokines (*CXCL10* and *CXCL5*); peptidase inhibition (*Serpina3n*); alteration in lipid/cholesterol synthesis [79]; and potentially, the disruption of blood-brain barrier integrity and function [80].

To add to the complexity of the astrocyte population, subtypes of reactive astrocytes have been identified in mice giving rise to the A1 (neurotoxic) vs. A2 (neuroprotective) classification [81], with ageing driving the predominance of A1 astrocytes (characterised by the expression of C3) in rodent brain [79] (Table 3).

**Table 3:** The different phenotypes acquired by the astrocytes

Phenotype	Protein (*gene*)	Function
Homeostatic/physiological status Human brain	GFAP Aldh1l1 S100B EAAT1, EEAT2 AQP4	Structural protein (intermediate filament) Formate oxidation Calcium-binding protein Glutamate transporters Water transporter
A1 (neuroinflammation “neurotoxic”) from rodent brain [79, 82]	Complement C3 (*C3*) Complement C1-inhibitor (*Serping*) GGTA1 (*GGTA1*) Interferon-inducible GTPase1 (*Ligp1*) Glycerol-1-phosphate phosphohydrolase 2 (*Gpp2*) Fibulin-5 (*Fbln5*) FKBP5 (*Fkbp5*) PSMB8 (*Psmb8*) Serglycin (*Srgn*) Amigo2 (*Amigo2*)	Central role in complement activation Inhibition of the complement system (belongs to serpin superfamily) Glycosphingolipid biosynthesis Resistance to intracellular pathogens Glycerol biosynthesis Maintenance of the vessel wall after injury Role in immunoregulation and cellular processes involved in protein folding and trafficking Participate in the immunoproteasomes Mediator of granule-mediated apoptosis May contribute to signal transduction
A2 (ischemia “neuroprotective”) from rodent brain [79, 82]	Cardiotrophin-like cytokine factor 1 (*Clcf1*) Keratinocyte tranglutaminase (*Tgm1*) Pentraxin 3 (*Ptx3*, TNF-inducible gene 14 protein) S100A10 (*S100a10*, p11) Sphingosine kinase 1 (*Sphk1*) CD109 (*Cd109*) Cyclooxygenase-2 (*Ptgs2*, COX2) Epithelial membrane protein 1 (*Emp1*) Solute carrier family 10 member 6 (*Slc10a6*) Transmembrane 4 L6 family member 1 (*Tm4sf1*) B3GNT5 (*B3gnt5*) CD14 (*Cd14*) STAT3 (*Stat3*)	B cell activation Transglutaminase enzyme Activate the classical pathway of complement and facilitate pathogen recognition Transport of neurotransmitters (serotonin), receptor for tissue-type plasminogen activator Regulates proliferation and survival Involved in degradation of TGFβ receptor 1 Promote inflammation Cell migration and proliferation Sodium-dependent transporter Role in cell development, activation, growth, and motility Enzyme Co-receptor for the detection of bacterial lipopolysaccharide Essential for the differentiation of the TH17 Helper T cells

*Aldh1l1*, Aldehyde dehydrogenase 1 family member L1; *Amigo*, Amphoterin-induced protein 2; *AQP4*, Aquaporin 4; *B3GNT5*, Lactosylceramide 1,3-N-acetyl-beta-D-glucosaminyltransferase; *EEAT*, Excitatory amino-acid transporter; *GFAP*, Glial fibrillary acidic protein; *GGTA1*, Alpha-1,3-galactosyltransferase; *GPP2*, Glycerol-1-phosphate phosphohydrolase 2; *FKBP5*, FK506 binding protein 5; *PSMB8*, Proteasome subunit beta type-8 (20S proteasome subunit beta-5i); *STAT3*, Signal transducer and activator of transcription 3

## Astrocytes in Alzheimer’s disease

Astrocytes participate in the neuroinflammatory processes by their properties to react to a range of pro- and anti-inflammatory components [83]. Both microglia and astrocytes have roles in the phagocytosis of cell debris and Aβ, and in responding to damage [84]. In addition, astrocytes participate in neuronal metabolic support as evidenced by preclinical studies [85]. In primary cultures of astrocytes, Aβ induces increased glucose uptake [86], altering their metabolic phenotype. This resulted in increased astrocytic MAO-B expression [87], one of the molecular targets to image astrocytes. In a transgenic mouse model of AD, astrocytic MAO-B overexpression caused excess gamma-aminobutyric acid (GABA) and excitotoxic glutamate, modifying homeostasis and leading to cognitive deficits [88].

In humans, clusters of reactive astrocytes around Aβ plaques are a hallmark of AD [89, 90]. However, the spatial distribution of astrocytes is independent of plaque size, different from microglia whose number around plaques correlates to plaque size [90]. Neuropathological studies have demonstrated increased reactive astrocytes in the vicinity of plaques as the disease progresses, but are unclear in determining whether astrocytes reaction is an early event [91, 92] or whether the cells play a role in the late stage of disease when dementia develops [93]. Nevertheless, imaging and *post-mortem* human studies have started to provide some clues on the dynamics of the astrocytic response in AD. GFAP expression is upregulated in AD [78], with A1 astrocytes forming the main population of the reactive cells in humans [82], consistent with a previous study highlighting variation in the pattern of GFAP, EAAT1, EAAT2, and S100B in relation to AD pathology [94]. Overall, evidence in the human brain supports astrocyte changes in AD as phenotypic rather than proliferative [95, 96], which has implications in terms of imaging.

## Molecular imaging targets for astrocytes

Multiple molecular imaging markers are needed to visualise astrocytosis as illustrated in Fig. 3, given that it is a highly dynamic process undergoing sequential protective (A2) and detrimental (A1) stages. There is increasing evidence that morphology and function may be inter-related in astrocyte activation, with an initial hypertrophic phase characterised by the overexpression of different markers involving MAO-B, intermediate filaments including nestin, vimentin, and GFAP [97] followed by an astrodegeneration phase characterised by atrophy, reduced branching and synaptic dysfunction, with reduced expression of markers such as Aquaporin-4 and glutamine synthetase.

### Enzymes

Among few astrocyte PET tracers available, ^11^C-deuterium-L-deprenyl (^11^C-DED) binds to MAO-B, an enzyme overexpressed in activated astrocytes [98]. Autoradiography studies have demonstrated that ^3^H-L-deprenyl binding partly overlaps with GFAP in AD and other neurodegenerative diseases [98–101], indicating a good level of specificity of MAO-B to activated astrocytes. Autoradiography data using ^11^C-L-deprenyl in AD brain tissue showed the highest tracer uptake at the earliest Braak stages, suggesting early involvement of astrocytosis in AD [102]. ^3^H-L-deprenyl had a different laminar pattern than that of Aβ deposition as measured by ^3^H-PiB [103], but partly co-located with tau as measured by ^3^H-THK5117 [104].

^11^C-DED PET imaging has been used to investigate astrocytosis in neurodegenerative diseases including AD [105, 106], amyotrophic lateral sclerosis, [107] and Creutzfeldt-Jakob disease [108]. Multitracer PET studies using ^11^C-DED, ^11^C-PiB, and ^18^F-fluorodeoxyglucose (^18^F-FDG) have allowed investigating the spatio-temporal patterns of in vivo brain astrocytosis, fibrillar Aβ deposition, and glucose metabolism at different stages of disease progression. In these studies, significantly increased ^11^C-DED binding was found in prodromal AD in comparison to healthy controls or AD dementia patients [106]. In autosomal-dominant AD, astrocytosis was observed at early presymptomatic stages using ^11^C-DED PET [109]; longitudinally, Aβ plaque deposition (^11^C-PiB) increased while astrocytosis (^11^C-DED) declined [110]. Early astrocytosis preceding Aβ plaque deposition was observed in transgenic AD mice [111].

### Receptors

The imidazoline-2 binding sites (I_2_BS) are located on the mitochondrial membranes of astrocytes [112] and are involved in the regulation of GFAP expression [113]. An increase in *post-mortem* density of I_2_BS has been observed in AD brain [114]. The novel PET tracer ^11^C-BU99008 has been characterised in preclinical species and demonstrated to be suitable to quantify cerebral I_2_BS density, as determined in rat [115], pig [116], and rhesus monkey [117]. More recently, ^11^C-BU99008 was found to have good kinetic properties in healthy human brain, with low affinity to MAO-A/MAO-B [118]. Studies in MCI and AD patients are underway to compare the regional distribution of ^11^C-BU99008 with that of Aβ and ^18^F-FDG-PET retention [119].

### Metabolic markers

^18^F-FDG-PET hypometabolism has been traditionally considered a biomarker for neuronal injury and neurodegeneration. However, a recent preclinical study showed evidence that astrocytes can contribute to ^18^F-FDG-PET as measured in healthy rat brain [120]. This study adds support to the astrocyte-neuron lactate shuttle hypothesis initiated 20 years ago, that suggests that neuronal energy demands are mostly met by lactate, originated in astrocytes and shuttled to neurons [121, 122]. Consistent with these preclinical findings, it was recently shown that longitudinal decline in astrocytosis, as measured by MAO-B expression, was correlated to progressive hypometabolism in autosomal-dominant AD mutation carriers [123], indicating that astrocytes may in part reflect metabolic activity in AD. The observed decline in MAO-B, potentially reflecting reduced glucose demand by astrocytes, might represent astrodegeneration, a glial phenotype characteristic of late stages of AD [97]. This link remains to be confirmed in a study associating ^18^F-FDG imaging of sporadic AD patients with human *post-mortem* examination.

Other potential targets have been explored in preclinical studies, but in vivo studies in human are still rare. For example, the astrocyte-specific glutamate transporters GLT1 (in rodents) and EAAT2 (in humans) were reduced in *post-mortem* tissue [124–126], suggesting loss of function of astrocytes at late disease stages. Similarly, glutamine synthetase was observed to decline with age in a transgenic mouse model of AD [127]. Another potential marker for astrocyte-related metabolic dysfunction is the impairment in GLUT1 (glucose transporter 1) protein expression, a glucose transporter predominantly expressed in astrocytes [128]. Interestingly, aerobic glycolysis, known to take place mostly in astrocytes, was observed to decline as tau accumulated in preclinical AD individuals [129], supporting astrocyte dysfunction as an early event in AD. These studies motivate the current research on PET imaging tracers that could target astrocyte-specific glutamate transporters in human brain including EAAT1/EAAT2 and GLAST (glutamate aspartate transporter). These studies will greatly contribute to understanding the contribution of astrocytes to the metabolic changes observed in AD.

## Emerging structural MRI and DTI imaging tools: Tracking morphological changes of activated astrocytes and microglia?

Microglia and astrocytes possess ramified morphologies, which under pathological conditions are modified towards a more ameboid-like (microglia) or hypertrophic-like (astrocytes) as described above. These morphological changes have motivated the development of structural magnetic resonance imaging (MRI) and diffusion tensor imaging (DTI) tools to incorporate into multimodal MRI/PET approaches that would better investigate the complex neuroinflammatory changes in AD. Using MRI and DTI, a recent study proposed a model of grey matter changes in AD [130], in which an early presymptomatic phase of decreased cortical mean diffusivity (MD) and increased cortical thickness (CTh), hypothesised to reflect hypertrophy or glial cell swelling due to neuroinflammation, is followed by increased cortical MD and decreased CTh reflecting neurodegeneration. More recently, the direct relationship between structural vs. inflammatory changes were tested in autosomal-dominant AD (Vilaplana et al., under review). In this study, ^11^C-DED binding had a negative association with cortical MD and a positive association with CTh in autosomal-dominant AD mutation carriers, suggesting that astrocyte activation and associated hypertrophy may explain the observed reduction in cortical MD and increases in CTh. Another recent study has found a positive correlation between microgliosis as measured by TSPO PET and grey matter volume at the early MCI stage of AD [131], suggesting that neuroinflammation is accompanied by cortical swelling from early stages. In the same lines, new analysis techniques for DTI imaging allow extracting a measure of cell body size called “cellular diffusivity” that is interpreted to reflect activated microglia or astrocytes [132]. These initial reports motivate further in vivo multimodal studies combining MRI, DTI, and PET imaging to fully characterise the complex molecular and morphological dynamics of neuroinflammation across disease progression in AD.

## Relationship between PET imaging of neuroinflammation vs. Aβ and tau proteinopathies

Recent preclinical evidence implies that glial activity could lead to tau deposition [133, 134]. It is still unclear whether the oligomeric forms of Aβ can cause the glial activation, which leads to tau formation and propagation throughout the cortex. Very few studies have imaged and compared inflammation and tau in human AD, and they support that inflammation is more strongly correlated to Aβ, likely preceding tau deposition [135–137]. However, longitudinal studies are needed to confirm these results.

## CSF and plasma biomarkers of neuroinflammation

One of the most promising and more extensively investigated CSF glial biomarker is YKL-40, a secreted glycoprotein expressed by microglia and astrocytes [138]. Recently, an increasing number of CSF biomarkers are being developed to track microglia and astrocyte activation, neuroinflammation, and cerebrovascular dysfunction including YKL-40, sTREM2, IL-6, IL-7, IL-8, IL-15, IP-10, monocyte chemoattractant protein (MCP)-1, intercellular adhesion molecule (ICAM)-1, vascular adhesion molecule (VCAM)-1, placental growth factor, and fms-related tyrosine kinase 1 (Flt-1) [139, 140]. CSF levels of YKL-40, ICAM-1, VCAM-1, IL-15, and Flt-1 were increased in AD already from the preclinical and prodromal stages and were associated with CSF tau especially in Aβ-positive individuals [140]. Interestingly, their increase was associated to cortical thinning. Studies comparing neuroinflammation CSF and PET biomarkers are to our knowledge still lacking, and thus, these studies will add very valuable knowledge in the near future.

## Neuroinflammation biomarkers and clinical trials

There is an urgent need to develop biomarkers for characterising multiple pathophysiological mechanisms including neuroinflammation [141]. Inflammation biomarkers are of interest in clinical trial design, due to their utility for patient stratification and to track biological effects of drugs. In this respect, the studies reviewed here motivate further research on the possible utility of neuroinflammation PET as well as structural MRI/DTI imaging measures, used alone or in combination, for their possible application as biomarkers in clinical trials. Consequently, it is essential to develop novel PET tracers that are more specific to astrocytes and to microglia, and that can selectively target their different inflammatory stages. Thus, translational studies i*n post-mortem* human brain are necessary to characterise these profiles and to develop a solid knowledge about what these tracers are binding to.

## Conclusion

To date, astrocytes have been investigated to a lesser extent than microglia for their role in AD; however, recent papers have emphasised the importance of a bidirectional communication between microglia and astrocytes via physical contacts and secreted molecules. Microglia, the sensors of changes in homeostasis, are the primary immune cells of the brain, which also regulate the immune functions of astrocytes. Experimental studies in diverse neurodegenerative diseases imply that microglia define the functions of astrocytes, ranging from neuroprotective to neurotoxic. Conversely, astrocytes appear to regulate microglial phenotypes and functions including motility and phagocytosis [92]. Of note, with this review we also emphasise the gaps between the markers identified in preclinical and human studies, the importance of phenotypes, and the choice of the imaging targets, highlighting the need for better translational research between these two areas.

Nevertheless, whether modulation of microglia/astrocyte cross-talk can ameliorate neurodegeneration in human AD remains to be demonstrated. Therefore, it is essential to increase our knowledge of the glial biology and of their interactions with the environment in a physiological/healthy context. This will identify targets to image glia which, whether in multimodal imaging studies or in combination with other parameters (fluid biomarkers), will clarify the involvement of glial cells in the course of the disease.

## Electronic supplementary material

ESM 1 (DOCX 124 kb)
