# Supplementary material for: Prospects and challenges of imaging neuroinflammation beyond TSPO in Alzheimer’s disease
Source: Eur J Nucl Med Mol Imaging. 2019 Aug 8;46(13):2831–47. doi: 10.1007/s00259-019-04462-w (PMC6879435; doi:10.1007/s00259-019-04462-w)
Supplement: Supplementary file 1 — (DOCX 124 kb) [file 259_2019_4462_MOESM1_ESM.docx]

*Faculty of the Multimodal Imaging in Neurodegeneration Cologne (MINC) symposium*

**Bénédicte Ballanger**, Lyon Neuroscience Research Center, Lyon, France, E.U.

**Henryk Barthel**, University Hospital Leipzig, University of Leipzig, Germany, E.U.

**Gérard N Bischof**, University Hospital Cologne, University of Cologne, Germany, E.U.

**Delphine Boche**, University of Southampton, Southampton, United Kingdom, E.U.

**Henning Boecker**, German Center for Neurodegenerative Disease (DZNE), Bonn Germany, E.U.

**Karl Peter Bohn**, University Hospital Cologne, University of Cologne, Germany, E.U

**Per Borghammer**, Aarhus University, Denmark, E.U.

**Donna Cross**, University of Utah, United States of America

**Donato Di Monte,** German Center for Neurodegenerative Disease (DZNE), Bonn Germany

**Alexander Drzezga,** University Hospital Cologne, University of Cologne, Germany, E.U., & Research Center Jülich, Germany, E.U.

**Heike Endepols,** University Hospital Cologne, University of Cologne, Germany, E.U.

**Kathrin Giehl,** University Hospital Cologne, University of Cologne, Germany, E.U.

**Michel Goedert,** Medical Research Council, Laboratory of Molecular Biology, Cambridge, United Kingdom, E.U.

**Jochen Hammes,** University Hospital Cologne, University of Cologne, Germany, E.U.

**Oskar Hansson,** Lund University, Sweden, E.U.

**Karl Herholz,** The University of Manchester, Manchester, United Kingdom, E.U.

**Günter Höglinger,** German Center for Neurodegenerative Disease (DZNE), Munich, Germany, E.U.

**Merle Hönig,** University Hospital Cologne, University of Cologne, Germany, E.U.

**Frank Jessen,** University Hospital Cologne, University of Cologne, Germany, E.U.

**Thomas Klockgether,** German Center for Neurodegenerative Disease (DZNE), Bonn Germany, E.U.

**Pierre Lafaye,** Institut Pasteur, Paris, France, E.U.

**Adriaan Lammertsma,** Amsterdam University Medical Center, Amsterdam, Netherlands, E.U.

**Eckhard Mandelkow,** German Center for Neurodegenerative Disease (DZNE), Bonn Germany, E.U.

**Eva-Maria Mandelkow,** German Center for Neurodegenerative Disease (DZNE), Bonn Germany, E.U.

**Andreas Maurer,** University Hospital Tübingen, Germany, E.U.

**Brit Mollenhauer,** University Medical Center Göttingen, Germany, E.U.

**Bernd Neumaier,** Research Center Jülich, Germany, E.U.

**Agneta Nordberg,** Karolinska Institutet, Stockholm, Sweden, E.U.

**Özgür Onur,** University Hospital Cologne, University of Cologne, Germany, E.U.

**Kathrin Reetz,** University Hospital Aachen, Germany, E.U.

**Elena Rodriguez-Vieitez,** Karolinska Institutet, Stockholm, Sweden, E.U.

**Axel Rominger,** University of Bern, Switzerland

**James Rowe,** University of Cambridge, Cambridge, United Kingdom, E.U. & Medical Research Council Cognition and Brain Sciences Unit, Cambridge, United Kingdom, E.U.

**Osama Sabri,** University Hospital Leipzig, University of Leipzig, Germany, E.U.

**Anja Schneider,** German Center for Neurodegenerative Disease (DZNE), Bonn, Germany, E.U.

**Antonio Strafella,** University of Toronto & Toronto Western Hospital, UHN, Toronto, Canada

**Stina Syvänen,** Uppsala University, Sweden, E.U.

**Thilo van Eimeren,** University Hospital Cologne, University of Cologne, Germany, E.U.

**Neil Vasdev,** The Centre for Addiction and Mental Health (CAMH), Toronto, ON, Canada

**Victor Villemagne,** Austin Health, Heidelberg, Victoria, Australia

**Dieter Willbold,** Research Center Jülich, Germany, E.U.
